# Decoding the Human Face: Progress and Challenges in Understanding the Genetics of Craniofacial Morphology

**DOI:** 10.1146/annurev-genom-120121-102607

**Published:** 2022-04-28

**Authors:** Sahin Naqvi, Hanne Hoskens, Franziska Wilke, Seth M. Weinberg, John R. Shaffer, Susan Walsh, Mark D. Shriver, Joanna Wysocka, Peter Claes

**Affiliations:** 1 Department of Chemical and Systems Biology, Stanford University School of Medicine, Stanford, California, USA; 2 Department of Genetics, Stanford University School of Medicine, Stanford, California, USA; 3 Center for Processing Speech and Images, Department of Electrical Engineering, KU Leuven, Leuven, Belgium; 4 Medical Imaging Research Center, University Hospitals Leuven, Leuven, Belgium; 5 Department of Biology, Indiana University–Purdue University Indianapolis, Indianapolis, Indiana, USA; 6 Department of Human Genetics, University of Pittsburgh, Pittsburgh, Pennsylvania, USA; 7 Center for Craniofacial and Dental Genetics, Department of Oral and Craniofacial Sciences, University of Pittsburgh, Pittsburgh, Pennsylvania, USA; 8 Department of Anthropology, University of Pittsburgh, Pittsburgh, Pennsylvania, USA; 9 Department of Anthropology, The Pennsylvania State University, University Park, Pennsylvania, USA; 10 Department of Developmental Biology, Stanford University School of Medicine, Stanford, California, USA; 11 Howard Hughes Medical Institute, Stanford University School of Medicine, Stanford, California, USA; 12 Department of Human Genetics, KU Leuven, Leuven, Belgium; 13 Murdoch Children’s Research Institute, Melbourne, Victoria, Australia

**Keywords:** craniofacial, development, genome-wide association study, GWAS, syndromes, gene regulation, phenotyping

## Abstract

Variations in the form of the human face, which plays a role in our individual identities and societal interactions, have fascinated scientists and artists alike. Here, we review our current understanding of the genetics underlying variation in craniofacial morphology and disease-associated dysmorphology, synthesizing decades of progress on Mendelian syndromes in addition to more recent results from genome-wide association studies of human facial shape and disease risk. We also discuss the various approaches used to phenotype and quantify facial shape, which are of particular importance due to the complex, multipartite nature of the craniofacial form. We close by discussing how experimental studies have contributed and will further contribute to our understanding of human genetic variation and then proposing future directions and applications for the field.

## INTRODUCTION

The human face and craniofacial structures exhibit a high degree of variation both among individuals of our own species and in comparison with those of other great apes (106). Changes in human craniofacial structures that occurred since the divergence from our closest living evolutionary relatives, chimpanzees and bonobos, facilitated the emergence of the uniquely human facial appearance and may have contributed to adaptations associated with bipedal locomotion, diet (79), enlarged brain size (114), and speech articulation (91). Consistent with the rapid evolution of human facial traits, modern human skulls display characteristic features that are distinct from those of extinct hominins such as *Homo erectus*, archaic humans, and Neanderthals (5), whereas other facial attributes (e.g., aspects of nose shape) have been postulated to mediate climate adaptation in human populations (174).

In addition to facilitating biological adaptations, the human face is a strong target of sexual selection and plays key roles in communication and other social interactions (53, 138). Craniofacial malformations—which are among the most common congenital diseases—therefore have not only huge medical implications but also devastating social consequences for affected patients and their families. Understanding the factors driving human craniofacial variation in health and disease is essential for comprehending what makes our faces both human and individual and for developing therapeutic strategies, treatment plans, and reconstruction techniques for a myriad of craniofacial disorders.

In this review, we discuss the contributions of genetics to understanding morphological craniofacial variation. We begin with a brief review of human craniofacial development, including some insights from studies of other species, before discussing studies that explore the overall heritability of human facial shape. Since the face is a complex morphological structure, we first discuss various phenotyping approaches and then provide an overview of craniofacial dysmorphism in rare syndromes. We next discuss genome-wide association studies (GWASs) of facial shape and associated insights into the genetic architecture of common variation, as well as the interplay between common and rare variation and associated conceptual models. Finally, we discuss post-GWAS analyses in the form of fine mapping and experimental functional studies before describing future possible directions for the field.

## THE DEVELOPMENT OF THE FACE

The development of the face involves a series of highly coordinated embryonic events, most of which occur between the third and eighth week of gestation (144). Tissues of the craniofacial complex are derived primarily from cranial neural crest cells (CNCCs), a transient population of cells that are specified from within the neural folds of the developing neural plate. Following specification, CNCCs undergo an epithelial-to-mesenchymal transition and migrate ventrally, eventually giving rise to most of the craniofacial skeleton and connective tissue through differentiation into cartilage, bones, and tendons (29). The first major division of developing craniofacial tissues occurs early in the fourth week of embryonic development, with the separation of CNCCs forming the frontonasal prominence from those populating the four pharyngeal arches. The frontonasal prominence later gives rise to the forehead and nasal bones (bridge and dorsum of the nose), while the first branchial arch gives rise to the paired maxillary and mandibular prominences, which at this point are still separated by the stomodeum (the precursor of the mouth). The remaining branchial arches give rise to noncraniofacial structures. In the fifth week, nasal placodes, from which the lateral nasal prominence and medial nasal prominence are derived, appear as thickenings on each side of the frontonasal prominence, and the mandibular prominences merge at the midline, contributing to the mandible, chin, and lower lip. Fusion of the medial nasal prominences is followed by fusion with the maxillary prominences approximately seven weeks after conception, leading to the formation of the central structures of the nose (columella) and central upper lip (philtrum). The sides and alae (wings) of the nose form when the lateral nasal prominences and the maxillary prominences begin to merge. These existing structures grow and mature during the remaining weeks of pregnancy (145).

The complex morphogenesis of the face, from the early specification and migration of CNCCs to the development of the facial prominences and derived structures, requires tightly controlled spatial and temporal signaling networks and gene expression patterns. Many of the mechanisms underlying early specification and morphological events have been uncovered by decades of study in model systems in which the early developmental transitions observed in human embryos are conserved, such as mouse and chick embryos. While a comprehensive review of such studies is beyond the scope of this work, two key concepts from this field emerge: first, that both cell-autonomous patterning of neural crest cells and embryo region-specific environmental cues by signaling ligands are key for proper craniofacial development, and second, that different combinations of the same signaling pathways pattern specific craniofacial regions in distinct ways. A major mechanism by which this patterning is achieved is by the combinatorial expression, through both cell-autonomous and signaling-responsive mechanisms, of sequence-specific transcription factors. The gene regulatory networks formed by these transcription factors have been delineated and reviewed in detail by Simões-Costa & Bronner (142). The major signaling pathways mediating environmental cues to CNCCs and other craniofacial cell types include fibroblast growth factor (FGF) (115), bone morphogenetic protein (BMP) (50), Sonic hedgehog (Shh) (41), wingless/Int-1 (128), and transforming growth factor beta (TGF-β) (40), all of which have been reviewed in detail elsewhere. Despite complex interactions between CNCCs and other cell types, such as the epithelium, in the formation of the craniofacial structures, cross-species transplantation experiments in birds [reviewed by Schneider (133)] have demonstrated that species-specific features of the craniofacial complex are driven largely by the neural crest, suggesting that CNCCs may in turn be the major factor determining variation in craniofacial morphology and risk for disease in humans, as discussed in subsequent sections.

Postnatally, the craniofacial complex undergoes continuous growth and remodeling, with the peak of growth occurring at puberty (145). The precise timing tends to be different for males and females; facial maturity develops between 12 and 14 years of age in females and approximately 2 years later in males (80, 105). Such remodeling is generally driven by the balance between osteogenesis, where osteoblasts form new bones and osteoclasts break down existing bone. These patterns of controlled formation and destruction are driven at least partly by biomechanical loading during normal function (43). The precise mechanisms by which these and other factors contribute to variation in remodeling remain largely unknown. Recently, however, studies have identified skeletal stem cells throughout the body (21, 22), and in the mouse lower jaw such skeletal stem cells give rise to osteoblasts in response to surgical bone separation, likely going through a neural crest–like intermediate state (126).

## ESTIMATES OF FACIAL FORM HERITABILITY

Facial variation is determined largely by genetic variation in combination with diverse environmental influences. The strong genetic component can be easily recognized and interpreted by perceptual observation; for example, there is a strong resemblance of facial features across families, and monozygotic twins are more alike than dizygotic twins. In addition, shared facial characteristics within ancestries (81) and the sexes (84, 105) as well as the distinctive facial features associated with particular genetic conditions (57) further demonstrate the extent to which genes influence craniofacial form and appearance.

Craniofacial heritability studies have been performed to estimate the proportion of the phenotypic variation that can be explained by genetics, as compared with environmental effects. Traditionally, this is done by measuring the facial similarity between relatives, such as twins (39, 154, 162) or parents and offspring (60, 73). The widespread availability of whole-genome microarrays and recent advances in statistical methods allow for these traditional family-based designs to be extended to large data sets of unrelated individuals where the degree of genetic relatedness is determined from genome-wide single-nucleotide polymorphisms (SNPs) (28, 171). However, these differences in study design, as well as differences in study populations and facial descriptors employed, have produced widely varying estimates of heritability in the literature, making direct comparisons across studies challenging. Research designs based on the similarity between twins or siblings tend to overestimate heritability due to shared environmental sources of covariance (common environment), whereas the lower bound is typically specified by the heritability estimate obtained from common SNPs using approaches that assume only additive effects (44). The acquisition of both genomic data and phenotypic measures for a sample of related individuals poses an interesting avenue to dissect this bias. Furthermore, heritability is a dynamic rather than a static descriptor, and estimates are expected to differ across populations and even within a single population due to varying genetic and environmental influences (158).

Despite these differences, strong genetic influences have predominantly been found for central midfacial parameters, with estimates exceeding 0.8 (i.e., >80% of variation is attributable to genetics) (27, 60, 111, 154, 162). In particular, high heritability has consistently been reported for aspects of nasal shape such as the position of the nasion, which is closely linked to the *PAX3* locus (1, 11, 19, 25, 61, 118, 164, 170). In addition to aspects of facial shape, facial size and allometry have been found to be highly heritable (28, 39). By contrast, stronger environmental contributions have been reported for the lower parts of the face, including the cheeks, mandible, and mouth, which are known to be affected by nutrition (141), aging (119), and oral function (123). Other nongenetic influences that can affect facial shape include prenatal exposure to teratogens [e.g., through maternal smoking (7) or alcohol intake (108, 150)], which may then lead to interactions with genes involved in craniofacial development, and the effects of climate (174), geography (151), and altitude (14) (e.g., through adaptation over evolutionary timescales).

## APPROACHES TO PHENOTYPING FACIAL FORM

Accurate and objective assessment of the human face, which is marked by inherent complexity and multidimensionality, is essential to any study focusing on craniofacial variation. Methods designed to measure aspects of facial form date back to at least the eighteenth century, with standardized anthropometric methods emerging by the early twentieth century (63). The field has grown exponentially since then through advances in acquisition techniques and analytical approaches.

The surface topography of the craniofacial complex is determined by two distinct but closely related components: the underlying facial skeleton, or hard tissue, and the overlying soft tissue facial structures (147). Compared with the skeletal component, the soft tissue component of the face is affected more by parameters such as age and body mass index (86, 141). However, these changes are unlikely to carry a strong craniofacial genetic component, and their effects are fairly predictable, so they can be controlled for statistically by means of regression in studies focused on the genetics of craniofacial variation.

Various imaging systems have been used to capture the facial surface and/or underlying skeleton in two or three dimensions (136). Three-dimensional imaging techniques are preferable because they successfully preserve the multidimensional nature of facial morphology. In facial genetic research today, approaches based on stereophotogrammetry and laser scanning are commonly used to generate high-quality images of the three-dimensional facial surface. Different acquisition systems are commercially available, all of which are comparable in terms of accuracy, with error rates well under 1 mm, as reviewed elsewhere (120). However, caution is needed when combining data obtained from different imaging systems into a single analysis in order to avoid systematic bias (165). An important drawback of three-dimensional imaging systems is that they are more expensive than their two-dimensional counterparts and are not always portable, although this is now beginning to change. Two-dimensional descriptions (1, 11, 19) and even categorical scoring of facial features (1, 121) remain valuable, especially in the context of large-scale data collection, but with the introduction of the TrueDepth camera in the iPhone X, three-dimensional facial imaging is becoming more accessible to the larger public (131). In medical imaging, cephalometry can be used to assess facial structures in two dimensions, while devices such as computed tomography and cone-beam computed tomography can preserve the three-dimensional structure of the underlying craniofacial skeleton in addition to the facial surface (136). However, radiation exposure generally limits their availability to clinical settings. By contrast, magnetic resonance imaging is a noninvasive technique that is directly informative about facial soft tissue structures, and it has been adopted in large-scale projects such as the UK Biobank (148) and the Adolescent Brain Cognitive Development (ABCD) study (17). The disadvantages of magnetic resonance imaging are its high cost and long acquisition times.

Landmarks can be placed on two- and three-dimensional representations of a face from which various quantitative measurements or features can be derived. Early approaches did so manually, focusing on well-defined anatomical points, but extensive effort has been put into the development of automatic methods to obtain fast, accurate, and reproducible results [4, 36, 90; for an in-depth overview of different landmarking approaches, we refer readers to a paper by Böhringer & de Jong (10)]. In addition, automatic methods allow for sparse descriptions to be expanded toward spatially dense configurations of quasi-landmarks covering the entire facial surface (26, 66). Here, the locations of the quasi-landmarks do not have any biological meaning per se, but homology is implied by each quasi-landmark that occupies the same position on the face relative to all other quasi-landmarks. Landmark configurations can subsequently be represented as linear distances, angles, and ratios measured between the landmarks (traditional anthropometrics), or the landmark *x-y-z* coordinates themselves can be analyzed [geometric morphometrics (175)]. The latter approach relies on superimposition methods to place the landmark configurations into a common frame of reference (56).

Of primary interest in facial genetics is the variation within and among subjects. Dimension reduction techniques such as principal component analysis are frequently applied to decompose the data into meaningful features while decreasing imaging noise. Indeed, the main modes of variation in the data are effectively described by the first several principal components following principal component analysis, and these modes have been analyzed separately or conjointly in genetic association studies (25, 27, 93, 96, 118, 125, 164). Moreover, quantitative assessment of variation in global facial shape has been complemented by the study of local features through the hierarchical partitioning of facial shape into smaller segments using machine learning approaches (25). Alternatively, prior biological knowledge can be integrated to guide the facial features extracted [e.g., by exploiting the facial resemblance within families (61)], or a lower-dimensional space can be built using deep learning methods. Deep learning with neural networks is widely used in biomedical image analysis (3), although it is fairly new to craniofacial genetic research, and most applications still utilize two-dimensional data. For example, Face2Gene, a popular tool developed by FDNA (Sunrise, Florida) to aid clinicians in the diagnosis of genetic disorders, applies deep learning algorithms to two-dimensional photographs of patients to learn relevant facial features for syndrome classification (54). Deep learning extensions for 3D surface or mesh data, referred to as geometric deep learning, are currently being developed (13, 101). However, the main challenge of deep learning remains the large data burden. In addition, techniques are often tailored to solve specific applications [e.g., facial classification (46)], and it is still unclear whether the learning outcomes are also relevant biologically.

## RARE VARIATION IN CRANIOFACIAL MORPHOLOGY

It has long been appreciated that craniofacial development can be severely disrupted. Since the early twentieth century, clinicians have been defining such syndromes on the basis of their distinctive phenotypes, which often involve features beyond the face. Craniofacial syndromes are individually rare but collectively common, with more than 500 clinical syndromes recognized (143). Furthermore, while each syndrome has a distinct constellation of features, some are often overlapping, including cleft lip and palate, craniosynostosis, and micrognathia.

Soon after the formalization of craniofacial syndrome diagnoses (37), and sometimes even alongside them (31), it was observed that such syndromes are often inherited in a Mendelian fashion, providing the first evidence for their genetic basis. At the same time, cases sometimes arose sporadically with no family history. Both types of cases would later be useful for gene mapping efforts starting in the 1980s; for example, mapping the translocation breakpoints in sporadic cases showed that *GLI3* and *SOX9* cause, respectively, Greig cephalopolysyndactyly syndrome and campomelic dysplasia (which includes craniofacial malformations, referred to as Pierre Robin sequence) (159, 160). By contrast, *TCOF1* and *FGFR2* were mapped as causative for Treacher Collins and Crouzon syndromes, respectively, largely through linkage analysis in family pedigrees (38, 127). Such gene mapping efforts were greatly accelerated by the development of Sanger sequencing and, later, massively parallel high-throughput approaches that allowed whole-exome and whole-genome sequencing. As with many studies of rare variation, and in contrast to studies of common variation described below, gene mapping was greatly simplified by the protein-coding nature of most causative mutations.

**Figure 1:**
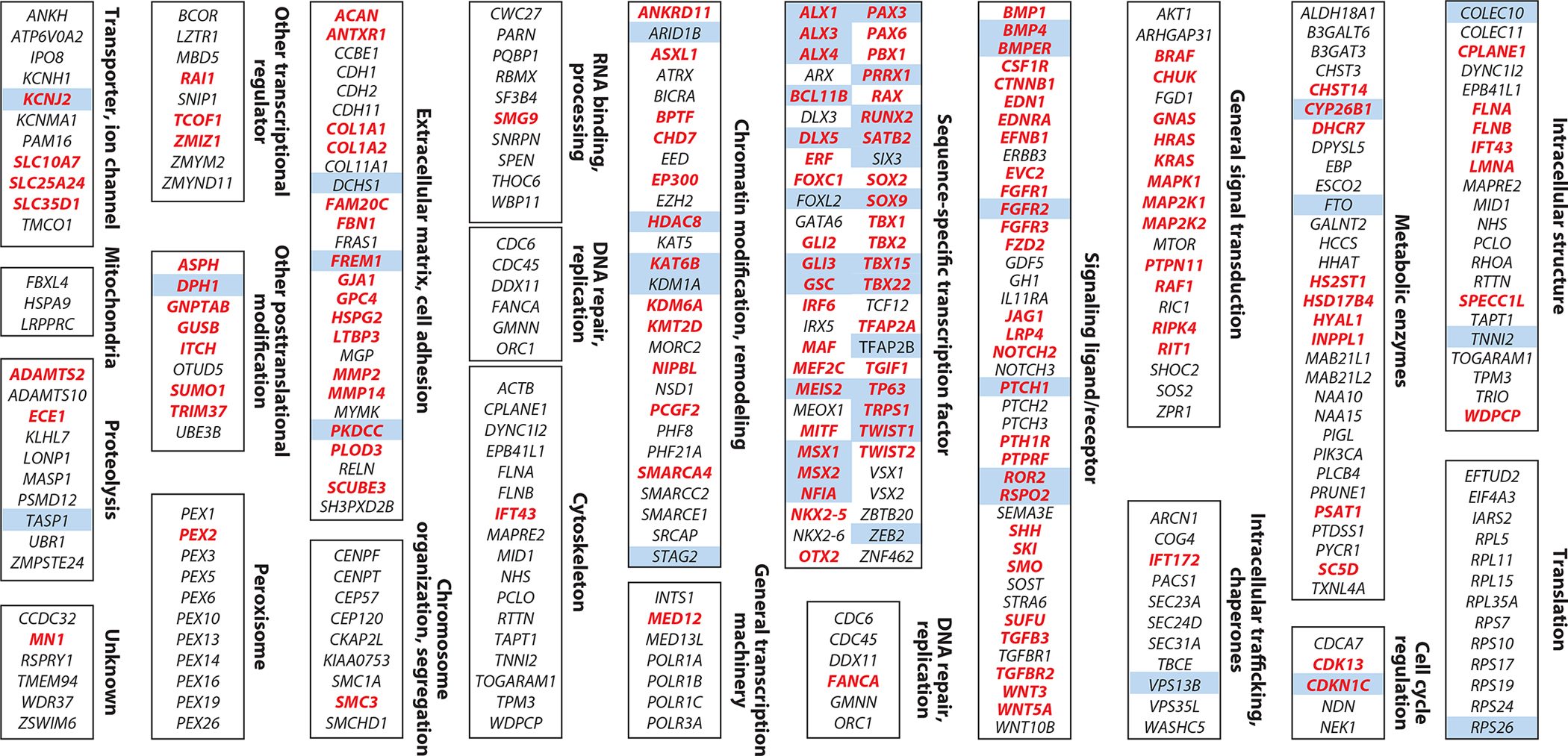
Genes and pathways mutated in rare craniofacial disorders with Mendelian inheritance. The genes were identified by curating hits for the search term craniofacial from the OMIM database, which were then organized into broad functional categories based partly on their PANTHER protein classes. Associations with GWASs were defined by aggregating the candidate genes and loci from studies listed in Table 1. Genes that cause craniofacial phenotypes when mutated in mice (*red*) were found by querying the Mouse Phenome Database. Blue shading indicates genes that GWASs have implicated in facial shape or nsCL/P. Abbreviations: GWAS, genome-wide association study; nsCL/P, nonsyndromic cleft lip with or without cleft palate; OMIM, Online Mendelian Inheritance in Man; PANTHER, Protein Annotation Through Evolutionary Relationship.

To gain a systematic understanding of the processes impacted in craniofacial syndromes, we searched the Online Mendelian Inheritance in Man (OMIM) database (58) for the term craniofacial and then manually reviewed each clinical synopsis to exclude erroneous results (i.e., when a lack of craniofacial syndromes was noted in the synopsis). This resulted in a list of 463 distinct clinical synopses involving craniofacial defects, which mapped to 343 distinct protein-coding genes (Figure 1; for further details, see Supplemental Table 1). Categorizing these genes into broad functional classes indicates that, while a wide range of molecular and cellular processes are perturbed in craniofacial syndromes, certain functions are more represented than others. One notable theme is the regulation of chromatin and transcription, both by sequence-specific transcription factors (the largest single group) and by enzymes that modify and/or remodel chromatin more broadly. Another prominent class is that of intracellular signaling ligands and their cognate receptors, along with secondary regulators of the same pathways. The prominence of sequence-specific transcription factors and signaling pathways can be understood in the context of the themes of craniofacial development discussed above, where different combinations of intracellular signaling ligands communicate environmental cues to cells of the developing face, in part through sequence-specific transcription factors, which are also in turn responsible for more cell-autonomous patterning of the craniofacial structures. General chromatin modifiers, on the other hand, are expected to have critical roles in the maintenance of transcriptional programs and cell identity in virtually all cells of the body, raising the question of why they are so prominent in craniofacial syndromes. One explanation is that some of these general regulators have additional, specialized functions that are particularly important for cells such as CNCCs (15); another is that CNCCs and their derivatives, along with other cell types that contribute to the face, are uniquely sensitized to these general perturbations of the chromatin and transcription machinery (52). Which of these two explanations is more common remains to be seen. Nevertheless, the presence of such coherent functional classes of genes and the large overlap with loci discovered by common variant association studies as well as mouse models (discussed in subsequent sections) illustrate the overall success of gene mapping rare craniofacial syndromes.

## COMMON VARIATION IN CRANIOFACIAL SHAPE

### Genetic Architecture

As discussed in the previous section, heritability studies of common facial variation have indicated a large genetic contribution, but the underlying genetic architecture remained relatively unknown until the advent of GWASs. While candidate gene studies have associated polymorphisms around a preselected gene of interest with various measures of facial shape (24, 68, 130), such studies do not provide insights into the overall distribution of variants affecting facial shape. GWASs test a panel of variants (typically SNPs) across the genome for association with a phenotype (here a facial shape measure); the first facial shape GWAS appeared in 2012. To date, there have been 25 GWASs of facial shape; they are summarized in Table 1, with additional details on loci from each study provided in Supplemental Table 2. As with most GWASs, the sample sizes started out small (several hundred individuals) and have since become much larger (tens of thousands of individuals), although these GWASs are not as large as many others for several reasons, including the limited availability of both genomic data and accurate facial scans from large numbers of individuals and the privacy issues inherent in obtaining such data on a large scale.

The number of loci discovered to date suggests that facial shape as a trait is highly polygenic in nature, but a more precise understanding of its genetic architecture requires quantifying the effect size of associations. This is something of a challenge for facial shape, as different studies have used different phenotyping approaches, some univariate and some multivariate. We aggregated all independent SNPs previously associated with facial shape from published GWASs (Supplemental Table 2) and used partial least-squares regression to estimate the fraction of variance explained by all such loci in a sample of 4,680 European-ancestry individuals (164). We found that 501 independent SNPs (*r*^2^ < 0.1), encompassing 303 loci, explained 13.7% of variance of the full face, represented by the first 36 principal components as determined by parallel analysis. In the same cohort, age, sex, and body mass index individually explained 7.0%, 12.2%, and 18.9% of the variance, respectively. These results are consistent with a polygenic architecture typical of complex traits, where many variants contribute individually small effects. As a comparison, for human height (one of the most polygenic complex traits), 3,290 independent, genome-wide significant variants explain 24.6% of variance (172). Despite having a polygenic architecture overall, a small number of common variants can have quite large effects on facial shape; a recent study specifically comparing the upper and lower tails of the distribution of facial shape (measured by specific principal components describing shape variation) found three variants with frequencies of approximately 10% and unusually large effects (odds ratios >4 for being in either tail) (30).

The two most common craniofacial birth defects, cleft lip with or without cleft palate (CL/P) and craniosynostosis, are an interesting point of contrast to the architecture of normal-range facial shape variation. As discussed above, both of these defects are found in a number of Mendelian craniofacial syndromes, but approximately 70% (74) and 50% (140) of CL/P and craniosynostosis cases, respectively, do not appear as part of a syndrome. Nonsyndromic CL/P (nsCL/P) occurs in 1 of every 920 live births in the United States (117), while the prevalence of isolated craniosynostosis has been estimated at between 1 in 2,100 and 1 in 2,500 live births (12, 87). There have been numerous GWASs of nsCL/P (6, 8, 35, 51, 64, 89, 98–100, 102, 103, 107, 149); these studies have been reviewed in detail elsewhere (152), but we also provide a combined list of variants and loci discovered by these studies in Supplemental Table 3. Together, these studies have demonstrated a substantial contribution of common variants to nsCL/P risk, with one study finding that approximately 30% of the variance in nsCL/P is attributable to all common SNPs (99). Notably, the same study found that the top 24 loci explain 25% of nsCL/P risk (99) in European-ancestry individuals, and another found that 26 loci explain approximately 11% of the variance in a Chinese cohort (173), indicating substantially larger effect sizes for nsCL/P than for facial shape variation. While nonsyndromic craniosynostosis common-variant GWASs have had less statistical power for variant discovery due to their smaller sample sizes, variants at the *BMP2* locus have been independently associated with two main subtypes, sagittal (76) and metopic (75) craniosynostosis. Here, too, the effect sizes of lead variants are unusually large relative to both facial shape variation and most common diseases (odds ratios >4 for sagittal and >2 for metopic craniosynostosis).

While the vast majority of GWASs to date have sought to discover variants with additive effects on the phenotype of interest, genetic interactions—either with other variants (gene × gene interactions, or epistasis) or with environmental variables—can be an additional source of variation. As a whole, GWASs of diverse phenotypes have found very few examples of large-effect epistatic interactions (129), and facial shape GWASs are similar in that regard. Several general explanations have been provided for this lack of epistasis, including a lack of statistical power to detect epistatic effects (as they are far smaller than the main additive effects) and the testing of tagging SNPs rather than the causal variant itself. Nevertheless, a few exceptions do exist. White et al. (164) found four pairs of loci, each with its own additive associations with distinct multivariate facial phenotypes, which show significant epistasis. Furthermore, Liu et al. (95) described an interaction between the *PRICKLE1* and *FOCAD* loci for cranial base width underlying a variance quantitative trait locus (QTL) at *PRICKLE1*. With respect to craniofacial disease, three distinct modifiers of nsCL/P subtypes have been discovered in the last few years: the *FAT4* locus for cleft laterality (34), the *PAX1* locus for bilaterality versus unilaterality (33), and a third locus for cleft palate (16).

Another important aspect of genetic architecture involves a description of genetic effects as a function of genetic ancestry. As with most GWASs to date, most facial shape GWASs have been performed in individuals of primarily European ancestry, making systematic comparisons across populations challenging. However, some notable studies in non-European individuals have included Latin Americans with admixed ancestry, Han Chinese individuals, and East African individuals (Table 1). While a direct comparison of phenotypic effects is somewhat challenging since the phenotype itself (i.e., the average facial shape) can be substantially divergent between populations, these studies have revealed that most of the variants affecting facial shape are shared between populations but a subset are likely population specific. This was clearest for East African individuals, which is unsurprising due to the higher divergence in allele frequency and linkage disequilibrium between African and non-African populations (93). Furthermore, a recent GWAS of nsCL/P risk from multiple broad ancestry groups found substantial dependence on ancestry in terms of SNP effect size and significance (109).

### Using Genome-Wide Association Studies to Understand Craniofacial Biology

In addition to describing genetic architecture (i.e., the number and effect sizes of variants associated with facial shape), GWAS approaches can also be used to unravel the biology underlying human facial variation in two related ways: first, by prioritizing specific cell types and *cis*-regulatory element classes important for variation, and second, by nominating candidate genes and pathways involved in the formation of the face.

As the vast majority of GWAS loci lie in noncoding regions of the genome, it follows that they lie in (or tag causal variants lying in) *cis*-regulatory elements controlling expression of coding genes. The cell types or tissues that are most enriched for GWAS-tagged regulatory elements would then be predicted to have important roles in trait variation. To date, maps of regulatory elements have been produced from both in vitro stem cell–derived CNCCs and their further derivatives (i.e., chondrocytes) (97, 122), as well as primary embryonic craniofacial tissue from Carnegie stages 12–20, during which time the craniofacial structures are still forming (167). The most straightforward and common approach uses a predefined set of facial GWAS loci (typically genome-wide significant SNPs) and either computes the amount of signal of a certain chromatin feature [such as acetylation of lysine 27 on histone H3 (H3K27ac)] within a set genomic distance of these variants or calculates the frequency with which the variants lie in regulatory elements defined by combinations of chromatin features. These enrichments or frequencies for the set of GWAS loci can then be compared with enrichments from a background set of loci not associated with facial shape to estimate the significance of enrichment. Such an approach has been applied to both normal-range facial GWAS loci (25) and loci associated with nsCL/P (99, 163) and has consistently found significant enrichment or overlap with active regulatory elements (mostly enhancers rather than promoters) in both in vitro–derived CNCCs and embryonic craniofacial tissue, as compared with other embryonic and adult human cell types and tissues that have been examined.

A newer, complementary type of approach incorporates genome-wide summary statistics along with patterns of linkage disequilibrium to compute an overall heritability enrichment of a GWAS for a specific cell type annotation (45). Although such an approach can have advantages over the simpler, cutoff-based approach, tools using it have generally been developed to work with highly powered GWAS and univariate traits, making it challenging to apply to facial shape GWASs, which are often multivariate in nature and use relatively small sample sizes. However, a recent study of the shared genetics of face and brain shape variation showed that the most widespread of these tools, linkage disequilibrium score regression, can be extended to the multivariate space with a few modifications. In addition to revealing a significant enrichment of face shape heritability in the in vitro–derived CNCCs, their chondrocyte derivatives, and embryonic craniofacial tissues, this approach showed that these cell types were not enriched for brain shape heritability (112). Overall, such studies suggest that much of the variation in facial shape arises from variants acting in a range of cell types during early embryonic development, although epigenomic maps of many of the human cell types and time points that are likely important to craniofacial development remain incomplete.

**Figure 2:**
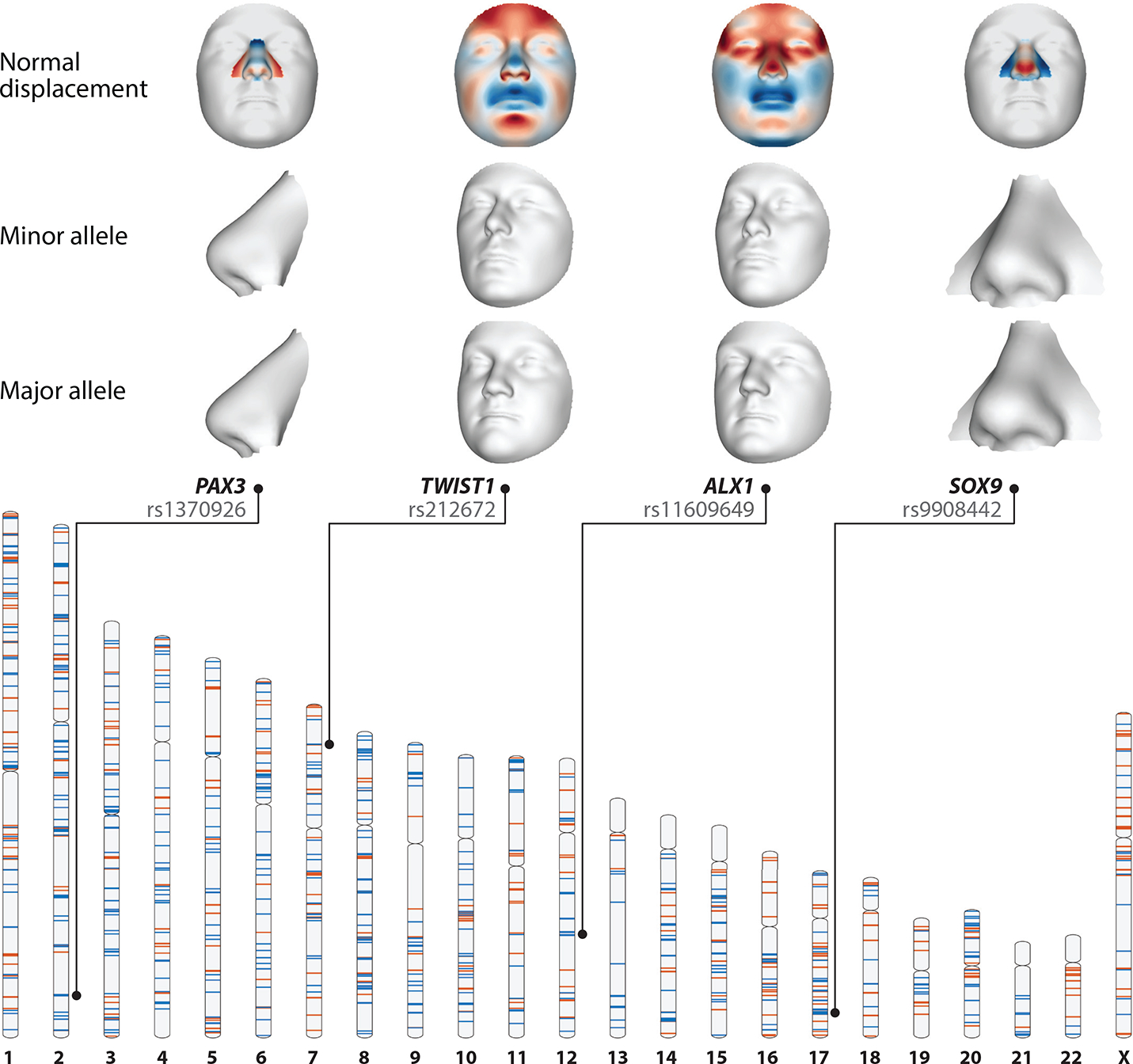
Genomic locations and regional effects of genome-wide significant loci for facial shape. In the ideogram, the genomic locations of lead SNPs identified through GWASs of facial shape in healthy individuals (listed in Supplemental Table 2) are shown in blue, and genes implicated in rare craniofacial disorders with Mendelian inheritance (listed in Figure 1) are shown in orange. Associated phenotypic effects of the *PAX3* (lead SNP rs1370926), *TWIST1* (lead SNP rs212672), *ALX1* (lead SNP rs11609649), and *SOX9* (lead SNP rs9908442) loci are illustrated by the facial morphs, exaggerated in the direction of the minor and major allele SNP variant based on the results of White et al. (164). Heat maps represent the normal displacement (displacement in the direction locally normal to the facial surface) in each quasi-landmark going from the minor to the major allele SNP variant. Blue indicates inward depression; red indicates outward protrusion. Abbreviations: GWAS, genome-wide association study; SNP, single-nucleotide polymorphism.

With respect to nominating candidate genes, one issue, which we discuss more below, is that both identification of the causal variant and assignment of the gene affected by the variant (since the vast majority of GWAS loci lie in noncoding regions) are far more challenging than they are for most craniofacial syndromes, which involve protein-coding variants. A common approach is to assign a GWAS-associated SNP to either the nearest gene or a gene within a fixed genomic distance that has known roles in craniofacial biology. While clearly heuristics, such approaches have revealed that many facial GWAS-associated loci contain genes implicated in rare craniofacial syndromes discussed above (Figure 2). For example, the first GWAS locus associated with common facial variation was *PAX3*, rare variants in which are known to cause Waardenburg syndrome. Additional candidate genes highlighted by GWASs include *SOX9*, *GLI3*, *TWIST1*, *ALX1*/*4*, *MSX1*, *TFAP2B*, *DLX5*/*6*, *FGFR2*, and *BMP2*/*4*. Indeed, of the craniofacial syndrome genes also highlighted by GWASs, there is a striking enrichment for sequence-specific transcription factors (Figure 1). There are several possible explanations for such an enrichment, ranging from the broad regulatory but cell type–specific roles of transcription factors to their inherent dosage sensitivity. Such hypotheses would be best addressed with genome-wide mapping of transcription factor targets as well as experimental measurements of their dosage sensitivities in the relevant cell types, which are yet to be done systematically.

## INTERPLAY BETWEEN COMMON AND RARE VARIATION

### Examples from Cleft Lip and Palate, Craniosynostosis, and Rare-Variant Studies of Healthy Individuals

Genetic studies suggest that both common and rare variation in craniofacial shape lie on the same continuum and share the same biological pathways, but to different extents: Common genetic variation results in subtle and cell type–specific changes in gene expression, largely by modulating activities of *cis*-regulatory elements such as enhancers, whereas rare variation results in larger changes in expression or activity of the same genes by impacting protein-coding sequence directly. One model to synthesize these observations is a liability threshold model, where a certain outcome (i.e., disease) occurs only after an individual passes a threshold due to the sum of multiple independent factors. The liability threshold model for disease has been appreciated for decades, but direct evidence that both common and rare genetic variation operate through the same pathways has emerged only in more recent years. For example, common genetic variants can modulate the risk of severe rare neurodevelopmental disorders, even though such disorders are largely monogenic and caused by rare (typically protein-coding) mutations (116). At the molecular level, common *cis*-regulatory variants have been observed to modify disease penetrance by either downregulating expression of rare pathogenic coding variants in healthy individuals or upregulating their expression in diseased individuals (18).

While most of the craniofacial disease burden is likely due to rare genetic variation, an interesting exception is CL/P, which, as discussed in the section titled Rare Variation in Craniofacial Morphology, is a phenotypic feature of many different craniofacial syndromes. However, a majority (approximately 70%) of CL/P cases are nonsyndromic, meaning that they occur in the absence of other clinical features. While nsCL/P may have a somewhat different genetic architecture from normal-range facial shape variation with respect to effect sizes, many loci are associated with both phenotypes. Studies explicitly testing the effects of nsCL/P risk loci on facial shape have found multiple loci with highly significant associations on both (9, 68). Indeed, one study found a significant association between a polygenic risk score for nsCL/P and the width of the philtrum, disruption of which leads to nsCL/P (62). Another recent GWAS of healthy, unaffected individuals, which used endophenotypes (intermediate phenotypes that can be used as markers for disease risk) derived from directly comparing facial scans of unaffected relatives of patients with nsCL/P with those of controls, found both overlap with the results of previous facial shape and nsCL/P GWASs and associations between polygenic nsCL/P risk scores and several endophenotypes (67). Thus, the case of nsCL/P suggests a liability threshold linking both normal-range and syndromic variation in facial shape.

If both common and rare variants operate along the same axis in craniofacial shape and disease, one would also expect epistasis between common and rare variation with respect to disease presentation, since common variants could move certain individuals away from or toward the threshold beyond which disease manifests. Exactly this type of interaction has been observed for nonsyndromic craniosynostosis, for which exome sequencing and analysis of de novo mutations in parent–child trios found a strong yet incompletely penetrant association with mutations in *SMAD6*, a negative regulator of BMP-dependent osteoblast differentiation (153). Remarkably, much of this incomplete penetrance was explained by a strong epistatic interaction with a common variant at the *BMP2* locus, which was discovered to have an independent effect in prior GWASs. Furthermore, this interaction occurred in the same pathway: BMP ligands bind and induce their cognate receptors to phosphorylate SMAD and activate transcription of osteogenic genes, a process directly inhibited by SMAD6.

Another test of a common axis between rare and common variants is to assess the impact of rare variants on facial morphology, but in individuals free of craniofacial disease. Rare variants are naturally expected to have larger effect sizes when detectable, and they can therefore bridge some of the gap between results from GWASs focusing on common variants and craniofacial disorders (syndromic or nonsyndromic). Few studies have assessed the impact of rare variants on facial shape due to the larger sample sizes required, but a recent study that focused on variants with <1% frequency and performed gene-based tests found seven genes to be enriched for rare variants affecting facial shape (94). One such gene, *NECTIN1*, has known roles in cranio-facial development and is associated with a syndrome (cleft lip/palate–ectodermal dysplasia syndrome), but, interestingly, the authors found no evidence of common variant associations within or near these seven genes. While this may suggest that rare variation can impact pathways discrete from those impacted by common variation, these low-frequency variants could still have epistatic interactions with common variants that could not be detected at the study’s sample size (2,329 individuals).

### A Multivariate Model Linking Craniofacial Shape Variation and Disease

**Figure 3:**
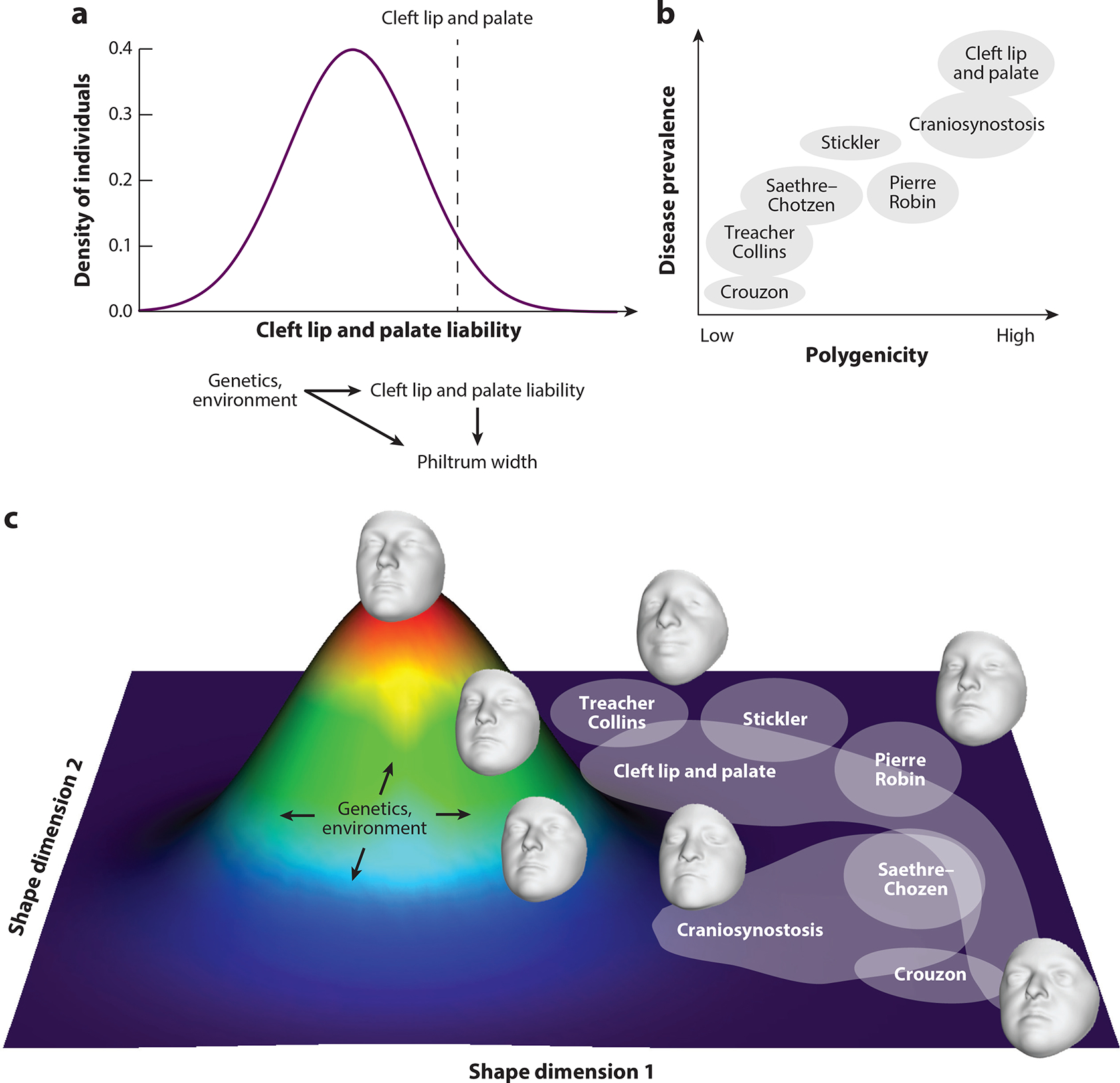
A multivariate model linking craniofacial shape and disease. (*a*) In standard liability threshold models, disease risk, which can be modulated by genetics or the environment, manifests itself as a univariate trait, or endophenotype (e.g., philtrum width for nonsyndromic cleft lip and palate). (*b*) Craniofacial disorders have a range of genetic architectures, from highly polygenic (*right*) to monogenic with largely Mendelian inheritance (*left*). (*c*) In the proposed multivariate shape space model, variation along multiple axes of shape (only two of which are shown for visualization purposes) leads to distinct yet overlapping zones of disease in the multidimensional shape space. The craniofacial syndromes are arranged approximately according to phenotypic similarity. Facial morphs for syndromes were created by first creating a univariate shape score distinguishing individuals with the syndrome from healthy controls and then moving four standard deviations along the univariate syndromic axis.

Studies to date have provided evidence of a liability threshold model for nsCL/P risk and specific univariate measures of facial shape, as well as clear epistatic interactions between rare and common variants for craniosynostosis (Figure 3a). But how do these findings generalize to the entirety of common facial shape variation, which GWASs have shown to be under multivariate genetic control, and craniofacial disorders, which range from the fairly prevalent and polygenic (such as nsCL/P and craniosynostosis) to Mendelian syndromes caused by one or a handful of genes (such as Treacher Collins and Crouzon syndromes) (Figure 3b), each of which has variable yet distinct facial features? We propose a description of craniofacial variation involving a multivariate shape space, a two-dimensional schematic of which is portrayed in Figure 3c. In this model, an individual lies at a point in high-dimensional space, the axes of which can be thought of as different aspects of shape variation. The distribution of individuals in this space can be thought of as a multivariate Gaussian distribution, and both genetic (common or rare) and environmental factors can move individuals around in this high-dimensional space. Different disease states occupy distinct but potentially overlapping zones in the space. For example, Mendelian craniofacial syndromes would occupy largely distinct zones due to their distinct facial characteristics, but nsCL/P and craniosynostosis would occupy a zone that overlaps multiple syndromes (i.e., Pierre Robin sequence, Treacher Collins syndrome, and Stickler syndrome for nsCL/P and Saethre–Chotzen and Crouzon syndromes for craniosynostosis). Such a model can also accommodate the variable expressivity observed in many Mendelian syndromes, since additional factors, such as an individual’s polygenic background or developmental environment, could shift them away from the center of the shape space zone corresponding to that syndrome.

## FUNCTIONAL STUDIES

There have been relatively few experimental functional studies directly based on findings from human genetic studies of craniofacial morphologies, in part because it can be challenging from both an ethical and practical perspective to re-create much of human craniofacial development in a dish. However, developments in human stem cell differentiation approaches, greater access to primary tissues from early developmental time points, and the use of mouse models have begun to provide some insights in this relatively nascent field. Here, we highlight some of the major objectives of such studies and a few examples.

### Experimental Fine Mapping and Elucidating Target Genes

Due to linkage disequilibrium between variants throughout the genome, population-based association studies such as GWASs inherently highlight regions of the genome and sets of linked SNPs rather than exact causal variants underlying a trait. Numerous approaches, collectively termed fine mapping, have been used to further narrow down candidate causal variants from GWASs. These include cross-ancestry GWASs, where distinct patterns of linkage disequilibrium between populations can narrow down candidate variants (assuming the same causal variant underlies variation in both populations); targeted fine-mapping GWASs, with denser panels of variants that allow for imputation of smaller linkage blocks; and purely computational approaches. These statistical approaches have been reviewed in detail by Schaid et al. (132). Here, we focus on fine-mapping approaches based on experimental data.

Experimental fine-mapping approaches vary in both the strength of evidence provided and scalability. Perhaps the most straightforward is to generate an epigenomic map from a cell type or tissue thought to have an important role in the trait of interest, with the rationale that associated variants located in regulatory regions are active in the cell type of interest. This approach has been widely used by several GWASs of face shape and/or disease (25, 99, 163, 164), due in part to its relative ease: Once epigenomic maps have been generated from sufficient cell numbers in the relevant ex vivo– or in vitro–derived cell type, they can be reused in future GWASs, and the fine mapping can be performed genome-wide. Stronger evidence can be provided by perturbing the activity of candidate regulatory elements harboring associated variants and assessing the impact on the expression of nearby genes. Yet stronger evidence results from re-creating the precise (typically single-nucleotide) changes of each trait-associated variant and assessing their impact on regulatory element activity and target gene expression.

Several studies have used experimental fine-mapping approaches beyond epigenomic maps. Xiong et al. (170) carried out a GWAS of univariate facial distances from multiple ancestries and performed both epigenomic fine mapping and luciferase reporter assays of resulting candidate SNPs in neural crest cells, finding significant effects for four out of five SNPs tested. Two recent in-depth studies of noncoding mutations underlying craniofacial syndromes provide further examples of what can be accomplished. Long et al. (97) combined epigenomic maps, enhancer reporter assays, and endogenous deletion of enhancers in both in vitro–derived CNCCs and mice to show that structural variants causing Pierre Robin sequence disrupt enhancers more than 1 Mb away from *SOX9*. Hirsch et al. (59) used similar approaches to demonstrate that structural variants encompassing parts of *HDAC9*, a neighboring gene to the craniofacial transcription factor *TWIST1*, cause craniosynostosis through disruption of *TWIST1* enhancers. These studies were in part successful at fine mapping the regulatory elements underlying disease-causing mutations because of their relatively large effect size, but this becomes more challenging for variants associated with common variation in facial shape or risk for nsCL/P or craniosynostosis.

While both Long et al. (97) and Hirsch et al. (59) provided strong evidence for causality by re-creating variants through genome editing in their endogenous regulatory context, such an approach is both time and resource intensive, especially if performed in relevant model systems, such as human pluripotent stem cell–derived CNCCs and their derivatives, or mice. A set of attractive in-between options is provided by massively parallel reporter assays and their numerous variants, all of which involve transfection or transduction of a library of DNA constructs consisting of regulatory elements of interest upstream of a reporter gene with genetic barcodes, allowing for sequencing-based readouts of all regulatory element activity simultaneously [reviewed by Inoue & Ahituv (69)]. Thus, the effect of multiple candidate variants located in hundreds or thousands of regulatory elements can be simultaneously assayed. We envision that such an approach could be applied to the many loci already discovered by facial shape GWASs, and the most prominent hits could be followed up with more resource-intensive approaches, including re-creating the precise variants in their endogenous context.

Fundamentally, genetic studies map variants associated with a phenotype; thus, connecting variants to genes is a key component of understanding phenotypic variation. As with most GWASs of complex traits and common diseases, most of the lead hits from studies of common facial shape variation and disease lie in noncoding regions of the genome. Again, the gold standard for connecting variants to target genes is re-creating naturally occurring variants endogenously and assessing their impact on nearby target genes, but as with fine mapping, higher-throughput approaches are necessary for mapping the target genes of the hundreds of variants associated with facial shape or disease to date. One possible higher-throughput approach is the generation of molecular QTL data sets, which involve measuring molecular traits such as RNA or protein levels or splicing in tissue or cells from a population of individuals. Genetic variants in this population can then be associated with molecular effects on a specific gene, and if such QTLs overlap with trait-associated variants, the associated genes are candidates for mediating the variant–trait association. Although eQTL data sets have yielded some success in fine mapping and target gene identification for other traits, eQTLs can miss the relevant target genes for a number of reasons, such as assaying cell types that are not important for the trait or insufficient statistical power [reviewed by Umans et al. (155)]. Due to the limited availability of primary craniofacial tissue from the relevant time points (i.e., during embryonic development), a more attainable avenue may be to use induced pluripotent stem cells (iPSCs) from a population of individuals and differentiate them into the relevant cell types (i.e., CNCCs or their chondrocyte/osteoblast derivatives). Studies in iPSC-derived cardiomyocytes and neurons suggest that sample sizes of at least 20–80 individuals are required to detect eQTLs with moderately large effect sizes when performing parallel differentiations of iPSCs from different individuals combined with bulk RNA sequencing (134, 146). Recent innovative approaches based on pooled differentiations of iPSCs from many individuals, combined with single-cell RNA sequencing to assign cells to individuals based on genetic polymorphisms, can both increase power by reducing noise due to differentiation batch effects and allow for larger sample sizes to be assayed in fewer experiments (32, 71).

A complementary approach to population-based studies is to map the target gene of the regulatory element (i.e., the enhancer) in which candidate causal variants (identified by some fine-mapping approach) reside. Broadly, these methods are based on the idea that enhancers come into close physical proximity to the promoters of their target genes, a feature that can be mapped genome-wide by chromosome conformation capture assays. Recent work has integrated such assays with other epigenomic data sets (i.e., measures of chromatin accessibility and histone modifications) to predict target genes of enhancers (47), with appreciable precision in applications to GWASs of common diseases, such as inflammatory bowel disease (113). Pairing chromosome conformation capture with chromatin immunoprecipitation (HiChIP) of active enhancer marks such as H3K27ac has also been used to map target genes of SNPs associated with both autoimmune and cardiovascular disease (110) as well as with prostate cancer (48).

### Epigenomic Maps from Relevant Cell Types

As mentioned throughout this review, epigenomic maps from cell types relevant to craniofacial shape have many uses in understanding variation, both in nominating the cell types and developmental times most important for variation and in fine-mapping variants. Thus far, such data sets in humans have been generated primarily from in vitro pluripotent stem cell–derived CNCCs or from bulk tissue samples from a few primary embryonic craniofacial stages. Numerous additional cell types and developmental times are important for craniofacial development, as evidenced by mouse studies, and may also explain additional variation in craniofacial morphology. These cell types include, for example, facial ectoderm, which secretes morphogens such as Shh, important for patterning CNCCs during early development (41), as well as cell types important for later bone remodeling, such as osteoclasts and recently identified skeletal stem cells (21). As discussed in the introduction, cross-species chimeras suggest that CNCCs are the main drivers of species-specific craniofacial patterning, whereas surface ectoderm is more important for the species-generic pattern (133); such logic may also apply to within-species variation, but this remains to be seen. Ideally, cell types would be assayed at a range of developmental times from primary human samples, but this is challenging due to both the complex nature of such samples (i.e., containing many cell types) and their limited availability.

The first challenge can be addressed using single-cell epigenomic profiling technologies, which currently provide single-cell, genome-wide data at low depth, but when grouped by cell type provide depth similar to that of bulk assays (139). The overall lack of availability of primary samples (as well as the inability to use them for further functional tests, for obvious ethical reasons) is one that may be partially ameliorated by the increasing success of in vitro organoid modeling approaches to recapitulate aspects of early human development. For example, a recent study used micropatterned chips to achieve robust morphogenesis through folding and closure of the neural tube, starting from commonly used pluripotent stem cells (77). Remarkably, these in vitro–derived neural tubes gave rise to migratory neural crest cells, which could be expanded and profiled further.

### Mouse Models of Human Craniofacial Variation

While many aspects of human craniofacial development can be probed using the approaches described above, they clearly lack the ability to experimentally perturb the development of a true craniofacial structure in vivo. Thus, despite its evolutionary distance and clear difference in overall craniofacial shape from humans, the mouse remains the most accessible and relevant in vivo model for testing hypotheses arising from studies of human craniofacial variation. Indeed, of the 343 genes associated with a Mendelian craniofacial syndrome in OMIM, 151 (44%) have orthologous genes in mice that result in craniofacial phenotypes when mutated, as indicated by the Mouse Phenome Database (Figure 1). Overall, this statistic indicates a high degree of conservation of the pathways underlying craniofacial development between human and mouse. Furthermore, given that many of these mutant mouse models were created after gene mapping of specific craniofacial syndromes, it also further illustrates the success of Mendelian craniofacial genetics in uncovering important regulators of craniofacial development.

Mouse studies based on GWASs of facial shape variation or nsCL/P risk are more challenging than those based on the human genetics of rare craniofacial disorders. This is in large part because most GWAS loci reside in noncoding parts of the genome, which in general are not well conserved between human and mouse. Even when the orthologous noncoding region exists in the mouse genome, its sequence, regulatory activity, or relative contribution to the target gene expression is often poorly conserved. For example, in a study by Long et al. (97) of enhancers of *SOX9* underlying Pierre Robin sequence, deletion of the orthologous conserved enhancers in mouse resulted in relatively subtle changes in *Sox9* expression and lower jaw morphology, as compared with the large corresponding effects associated with the loss of human enhancers in CNCCs and patients. Indeed, a recent study of two genes highlighted specifically by facial shape GWASs, *Pax1* and *Tbx15*, resorted to mutating protein-coding sequence, as the candidate regulatory elements underlying the GWAS loci are not known with certainty and are in any case not well conserved in mouse (124). Similarly, Adhikari et al. (1) used gain- and loss-of-function mutants of *Edar*, SNPs near which were associated with chin protrusion in a Latin American cohort, to demonstrate an effect on mandible length.

One potential solution to the lack of noncoding sequence conservation between human and mouse would be to insert variants of the human regulatory element underlying a GWAS hit (determined using the experimental fine-mapping approaches discussed above) into the syntenic region of the mouse genome and then assess the resulting effects on target gene expression and cranio-facial morphology. However, it is possible, if not likely, that the regulatory landscape surrounding the mouse orthologous locus has diverged enough that the perturbation may not reflect regulatory changes equivalent to those influenced by the variant in the context of human development.

## FUTURE DIRECTIONS

The human face is a highly variable and multipartite structure resulting from coordinated action between diverse genetic and nongenetic factors. Recent advances in phenotyping strategies and analytical approaches have substantially improved our understanding of the genetic contributions to craniofacial morphology, and we expect this trend to continue in future years, though some challenges remain.

GWASs have illuminated the polygenic nature of craniofacial morphology, uncovering more than 300 genetic loci associated with one or multiple aspects of facial form. One of the major challenges continues to be the identification of the associated variants for function and the mechanisms by which they alter gene function or expression. In addition, individual variants contribute only a small proportion of the phenotypic variance, and a large number of genetic loci are yet to be discovered, since they likely have effect sizes that are too small or allele frequencies that are too rare to be detected with statistical significance using current sample sizes, which are still modest by modern GWAS standards. Furthermore, epistatic effects between combinations of variants acting on overlapping areas of the face shape space likely also have relatively small effects and thus remain undiscovered. A natural way to increase statistical power is to increase the size of the study cohort, for instance, through joint collaborative efforts. For traditional anthropometric measurements, this is relatively straightforward, but it becomes more challenging when principal components or multivariate descriptions of the face are used. The latter requires sharing individual raw images or the underlying principal component analysis constructs, which is often not possible due to strict privacy considerations.

Combining data sets is further complicated when individuals of diverse ancestral backgrounds or developmental stages are included. Although the need for increasing sample diversity in genetic studies is now widely appreciated, most facial shape GWASs have been performed in relatively homogeneous cohorts of primarily European descent. In cases of population heterogeneity, proper statistical considerations are required to control for population stratification. One approach is to stratify samples into major population groups, as determined by principal component analysis, and combine the results in a cross-ancestry meta-analysis. Alternatively, the joint analysis of the full cohort offers advantages in terms of statistical power for discovery (168) and its ability to generate ancestry-specific estimates (2). To deal with age as a confounder, studies have either analyzed individuals at a single developmental time point or removed the effects of age statistically. However, such corrections are inherently flawed, as facial growth and development and, later in life, facial aging are complex, nonlinear processes. Nonlinear kernel-based methods have been developed for three-dimensional facial data (104, 105), but the collection of longitudinal data will also offer unique insights into the modeling of craniofacial growth.

Larger cohorts of either healthy individuals or those with craniofacial disorders will also allow for a further blurring of the distinction between common and rare variation in genetic studies. For example, sufficiently large cohorts of patients with neurodevelopmental disorders allowed for the discovery of a common variant burden modulating disorder risk (116); in the context of craniofacial disorders, such an approach could be used to test whether distinct syndromes have correlated but not identical common variant burdens, as predicted by the multivariate liability threshold model. Large cohorts of healthy individuals would allow for the identification of individuals who have rare mutations in important craniofacial genes but no disease, which could reveal additional environmental or genetic factors that reduce disease penetrance. Larger sample sizes would also allow for the explicit modeling of gene–environment interactions, which remain underexplored with respect to facial variation.

As our knowledge of the genetic architecture of the human face grows, it will become possible to use this information in concrete, real-life applications. Perhaps one of the most appealing applications in human facial genetics is the prediction or reconstruction of facial features from biological material, for example, collected from patients [e.g., to guide orthodontic treatment or facial reconstructive surgery (72)], recovered from skeletal remains [e.g., to visualize the appearance of hominin ancestors (49)], or left at a crime scene [e.g., to narrow down potential suspects (23)]. Forensic DNA phenotyping, or the prediction of physical appearance from DNA, shows promise when comparative DNA profiling fails (i.e., when no match between the unknown sample and suspect or known profiles in a DNA database can be obtained) (82). The simultaneous prediction of eye, hair, and skin color has already been validated forensically (20). However, current prediction results for facial morphological traits do not reach high enough accuracy, and commercial tools that claim to do so lack any scientific validation (166). Furthermore, previous studies have shown that predictions are driven only by ancestry and sex (24, 92, 135), and complex patterns of interactions among genes and/or with the environment further complicate matters (55). Methods to predict facial appearance and infer DNA-related features have also raised concerns regarding privacy and ethics (78, 83, 156), although the actual risk of reidentification was recently shown to be smaller than previously claimed (157). The establishment of adequate legislation and regulatory frameworks for any practice or application will be a necessity for its implementation.

## Supplementary Material

Supplementary Tables 1-3

Supplementary Tables 1-3 Descriptions

## Figures and Tables

**Table 1:** GWASs of facial shape in healthy individuals

Study	Year	Study design	Ancestry	Phenotypes	Key findings
Paternoster et al. (118)	2012	Discovery (*N* = 2,185), replication (*N* = 1,622)	European	Linear distances (*N* = 54) and principal components (*N* = 14) derived from three-dimensional facial images	Identified genome-wide significant associations at 4 loci; replicated 1 signal; candidate gene *PAX3*
Liu et al. (96)	2012	Discovery (*N* = 5,388), replication (*N* = 4,435)	European	Linear distances (*N* = 36), principal components *(N* =11), and centroid size derived from three-dimensional head magnetic resonance imaging and two-dimensional facial images	Identified genome-wide significant associations at 5 loci; candidate genes *PRDM16*, *PAX3*, *TP63*, *C5orf50*, and *COL17A1*
Jacobs et al. (70)	2014	Meta-analysis (*N* = 6,631)	European	Graded categorical scoring of sagging eyelids (*N* = 1)	Identified genome-wide significant associations at 1 locus; candidate gene *TGIF1*
Pickrell et al. (121)	2016	Discovery (*N* = 70,000)	European	Diverse traits and diseases (*N* = 42), including the (graded) categorical facial features (*N* = 2) nose size and chin dimples	Identified genome-wide significant associations at 13 and 57 loci affecting nose size and chin dimples, respectively
Adhikari et al. (1)	2016	Discovery (*N* = 6,275), replication (*N* = 501)	Latin American	Linear distances and angles (*N* = 10) and graded categorical features (*N* = 14) derived from two- and three-dimensional facial images	Identified and replicated genome-wide significant associations at 6 loci; candidate genes *EDAR*, *PAX3*, *DCHS2*, *SUPT3H/RUNX2*, *GLI3*, and *PAX.1*
Shaffer et al. (137)	2016	Meta-analysis *(N* = 3,118)	European	Linear distances (*N* = 20) derived from three-dimensional facial images	Identified genome-wide significant associations at 7 loci; candidate genes *MAFB*, *PAX9, MIPOL1*, *ALX3, HDAC8*, and *PAX1*
Cole et al. (27)	2016	Discovery (*N* = 3,505), replication (*N* = 2,390)	African	Linear distances (*N* = 25), principal components (*N* = 6), and facial size measures (*N* = 3) derived from three-dimensional facial images	Identified and replicated genome-wide significant associations at 2 loci; candidate genes *SCHIP1* and *PDE8A*
Lee et al. (88)	2017	Meta-analysis (*N* = 2,817)	European	Factors of linear distances *(N* =23) derived from three-dimensional facial images	Identified genome-wide significant associations at 3 loci; candidate genes *PARK2* and *FREM1*
Crouch et al. (30)	2018	Discovery (*N* = 1,832), replication (*N* = 1,567)	European	Principal component extremes (*N* = 40) derived from three-dimensional facial images	Identified genome-wide significant associations at 27 loci; replicated 3 signals; candidate genes *PCDH1*5, *MBTPS1*, and *TMEM163*
Claes et al. (25)	2018	Discovery (*N* = 2,329), replication (*N* = 1,719)	European	Combined principal components representing global-to-local facial segments (*N* = 63) derived from three-dimensional facial images	Identified genome-wide significant associations at 38 loci; replicated 15 signals; candidate genes *TBX15*, *ASPM*, *PKDCC,* HOXD cluster, *PAX3, RAB7A/ACAD9, EPHB3*/*DVL3*, *DCHS2*, *SUPT3H, RPS12*/ *EYA4, DLX6/DYNC1L1*, *BC039327*, *SOX9,* and *KCTD15*
Endo et al. (42)	2018	Meta-analysis (*N* = 11,311)	East Asian	Diverse skin-related traits (*N* = 7), including the categorical scoring of double-edged eyelids	Identified genome-wide significant associations at 1 locus affecting double-edged eyelids; candidate genes *EMX2*, and *EMX20S*
Cha et al. (19)	2018	Discovery (*N* = 5,643), replication (*N* = 1,926)	East Asian	Linear distances, angles, areas, and curvature (*N* = 85) derived from two-dimensional facial images	Identified genome-wide significant associations at 5 loci; candidate genes *OSR1-WDR35*, *HOXD1-MTX2*, *WDR27*, *SOX9,*and *DHX35*
Howe et al. (62)	2018	Meta-analysis (*N* = 6,136)	European	Linear distance (*N* = 1) derived from three-dimensional facial images representing philtrum width	Identified genome-wide significant associations at 2 loci affecting philtrum width; candidate genes *HOXA1* and *ENSG00000232633*
Qiao et al. (125)	2018	Discovery (*N* = 1,709), replication (*N* = 1,675)	European, East Asian, admixed European-Asian	Linear distances (*N* = 10), principal components (*N* = 6), and partial least-squares components (*N* = 6) derived from three-dimensional facial images	Identified genome-wide significant associations at 6 loci; replicated 4 signals; candidate genes *COL23A1*, *PCDH7, UBASH3B*, and *BMP2*
Wu et al. (169)	2019	Discovery *(N* = 50)	East Asian	Linear distances (*N* = 48) derived from three-dimensional computed tomography scans of the skull	Identified genome-wide significant associations at 4 loci; candidate genes *RGPD3*, *IGSF3*, *SLC28A3*, and *USP40*
Xiong et al. (170)	2019	Discovery (*N* = 10,115), replication (*N* = 7,917)	European, Latin American, admixed European-Asian	Linear distances (*N* = 78) derived from three-dimensional facial images	Identified genome-wide significant associations at 24 loci; replicated 10 signals; candidate genes *CASZ1*, *INTU, KIF6*, *TBX3, C14orf64*, *RPGRIP1L*, *PAX3, SFRP2*, *ROR2,*and *PAX1*
White et al. (164)	2021	Meta-analysis (*N* = 8,246)	European	Combined principal components representing global-to-local facial segments (*N* = 63) derived from three-dimensional facial images	Identified genome-wide significant associations at 203 loci and study-wide significant associations at 120 loci; found 117 new and 86 previously identified signals
Bonfante et al. (11)	2021	Discovery (*N* = 6,169)	Latin American	Linear distances, ratios, and angles (*N* = 59) derived from two-dimensional facial images	Identified genome-wide significant associations at 32 loci; replicated 23 signals and found 9 new signals
Indencleef et al. (67)	2021	Meta-analysis (*N* = 8,246)	European	Scores representing nsCL/P endophenotype (*N* = 59) defined across global-to-local facial segments (*N* = 63) derived from three-dimensional facial images	Identified genome-wide significant associations at 29 loci and study-wide significant associations at 9 loci; 22 signals were previously identified in GWASs on normal-range facial variation, and 18 signals were near genes with strong evidence in orofacial clefting
Huang et al. (65)	2021	Discovery (*N* = 2,659); additionally tested 56 SNPs previously reported in facial GWAS literature	East Asian	Combined principal components representing different facial segments (*N* = 10) derived from three-dimensional facial images	Identified genome-wide significant associations at 7 loci and study-wide significant associations at 4 loci; replicated 24 SNPs from previously reported genetic loci near *DCHS2*, *SUPT3H*, *HOXD1*, *SOX9, PAX3*, and *EDAR;* candidate genes *DENND1B*, *PISRT1, DCHS2/ SFRP2*, and *VPS13B*
Liu et al. (94)	2021	Discovery (*N* = 2,329); tested low-frequency coding variants (MAF < 1%) in 8,091 genes	European	Combined principal components representing global-to-local facial segments (*N* =31) derived from three-dimensional facial images	Identified significant associations at 7 genes: *HFE, NECTIN1, CARS2*, *LTB4R, TEL02,AR,* and *FTSJ1*
Hoskens et al. (61)	2021	Meta-analysis (*N* = 8,246)	European	Scores representing facial traits shared by siblings (*N* = 1,048) defined across global-to-local facial segments (*N* = 63) derived from three-dimensional facial images	Identified genome-wide significant associations at 218 loci and studywide significant associations at 38 loci; found 109 new and 109 previously identified signals
Liu et al. (93)	2021	Discovery (*N* = 2,595)	African	Combined principal components representing global-to-local facial segments (*N* = 63) derived from three-dimensional facial images	Identified genome-wide significant associations at 20 loci and study-wide significant associations at 6 loci; 10 signals were shared with Europeans
Wang et al. (161)	2021	Meta-analysis (*N* = 9,674)	East Asian	Combined principal components representing global-to-local facial segments (*N* = 63) derived from three-dimensional facial images	Identified genome-wide significant associations at 244 loci and study-wide significant associations at 151 loci; found 130 new and 114 previously identified signals; 89 signals were shared with Europeans
Knol et al. (85)	2021	Meta-analysis (*N* = 34,130), replication (*N* = 10,115)	European	Interorbital distance derived from magnetic resonance imaging	Identified genome-wide significant associations at 56 loci; 46 signals were new for facial morphology

Abbreviations: GWAS, genome-wide association study; MAF, minor allele frequency; nsCL/P, nonsyndromic cleft lip with or without cleft palate; SNP, single-nucleotide polymorphism.
