# Supplementary material for: Decoding the Human Face: Progress and Challenges in Understanding the Genetics of Craniofacial Morphology
Source: Annu Rev Genomics Hum Genet. Author manuscript; Available in PMC 2022 Sep 18. (PMC9482780; doi:10.1146/annurev-genom-120121-102607)
Supplement: Supplementary Tables 1-3 Descriptions [file NIHMS1833977-supplement-Supplementary_Tables_1-3_Descriptions.pdf]

Supplemental Table descriptions:

Supplemental Table 1. Genes and pathways mutated in rare craniofacial disorders with Mendelian inheritance. Genes were identified by searching the Online Mendelian Inheritance in Man (OMIM) database for the term 'craniofacial,' and then manually reviewing clinical synopses to confirm a craniofacial defect in the accompanying syndrome. Genes implicated by GWAS were identified through literature review of all facial shape and nsCL/P GWAS (Supplementary Tables 2 and 3). Genes for which mouse mutants have craniofacial phenotypes were identified by searching the Mouse Genome Informatics database (MGI; http://www.informatics.jax.org/allele) for the phenotype term 'craniofacial' and further screening entries for loss-of-function mutations.

Supplemental Table 2. SNPs associated with normal-range facial variation through GWAS. SNPs as well as corresponding candidate genes were collected from previously published GWAS.

Supplemental Table 3. SNPs associated with orofacial clefts through GWAS. SNPs as well as corresponding candidate genes were collected from previously published GWAS.
